# A Comprehensive Review of Muscle–Tendon Junction: Structure, Function, Injury and Repair

**DOI:** 10.3390/biomedicines12020423

**Published:** 2024-02-12

**Authors:** Siqi Tong, Yuzhi Sun, Baian Kuang, Mingyue Wang, Zhixuan Chen, Wei Zhang, Jialin Chen

**Affiliations:** 1 School of Medicine, Southeast University, Nanjing 210009, China; 2 Center for Stem Cell and Regenerative Medicine, Southeast University, Nanjing 210009, China; 3 Department of Orthopaedic Surgery, Institute of Digital Medicine, Nanjing First Hospital, Nanjing Medical University, Nanjing 210006, China; 4 Jiangsu Key Laboratory for Biomaterials and Devices, Southeast University, Nanjing 210096, China; 5 China Orthopedic Regenerative Medicine Group (CORMed), Hangzhou 310058, China

**Keywords:** muscle–tendon junction, dystrophin, α7β1 integrin, MTJ repair, MTJ tissue engineering, scaffold

## Abstract

The muscle–tendon junction (MTJ) is a highly specific tissue interface where the muscle’s fascia intersects with the extracellular matrix of the tendon. The MTJ functions as the particular structure facilitating the transmission of force from contractive muscle fibers to the skeletal system, enabling movement. Considering that the MTJ is continuously exposed to constant mechanical forces during physical activity, it is susceptible to injuries. Ruptures at the MTJ often accompany damage to both tendon and muscle tissues. In this review, we attempt to provide a precise definition of the MTJ, describe its subtle structure in detail, and introduce therapeutic approaches related to MTJ tissue engineering. We hope that our detailed illustration of the MTJ and summary of the representative research achievements will help researchers gain a deeper understanding of the MTJ and inspire fresh insights and breakthroughs for future research.

## 1. Introduction

It is widely acknowledged that the muscle–tendon junction (MTJ) serves as the pivotal conjunction between muscle and tendon, facilitating the force transmission from the muscle to the tendon. MTJ injury is often accompanied by muscle and tendon injuries, resulting in restricted force transmission [1]. At the MTJ, the connective tissue surrounding the muscle is seamlessly integrated with that within the tendon. This integration includes the epimysium and epitenon, perimysium and endotenon, as well as endomysium. These continuous structural elements are responsible for lateral force transmission, which is achieved via connections from the sarcomere to the sarcolemma through to the costamere [2]. This force is then further transmitted from the sarcolemma to the lamina densa, endomysium, and perimysium, aided by collagen fibers [3]. The definition of the MTJ remains a topic of debate, with ongoing discussion regarding whether it represents a gradual transition zone or a distinct boundary between muscle and tendon. Nevertheless, it is evident that the MTJ cannot be clearly defined through gross observation. Clinical studies on MTJ injuries, such as sprains, partial tears, and complete ruptures, often provide vague descriptions of this critical anatomical region [4,5]. A precise characterization of the MTJ requires a profound exploration of its microstructural and submicrostructural components, elucidating the structural–functional relationship. Such an understanding is pivotal for advancing knowledge of MTJ-related diseases.

Since the MTJ plays a significant role in the musculoskeletal system and bears substantial stress during sports activities, the MTJ is highly vulnerable [6]. Due to its highly specialized structure, spontaneous repair is hampered after injury, which brings about enormous challenges to MTJ repair [6]. Current conservative treatments are primarily effective for minor MTJ sprains, while partial tears and complete ruptures often require surgical suturing, although outcomes can be suboptimal [7]. The continuous development of tissue engineering has brought about original opportunities for MTJ repair [8]. MTJ tissue engineering is based on the preparation of scaffolds assembled with different types of cells and growth factors that should support tissue regeneration [9]. However, the complete restoration of tissue function remains a formidable challenge [9].

## 2. The Structure and Definition of MTJ

**Figure 1:**
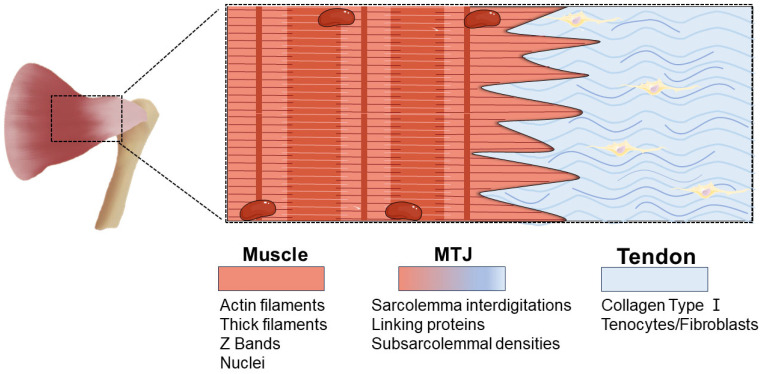
Schematic diagram of the adult MTJ interface (sagittal section). Tendon-produced collagen fibers are anchored vertically to finger-like protrusions of the MTJ membrane. The sub-membrane density at the tip of the finger-like projection corresponds to the muscle side of the MTJ. These densities are generated due to a substantial accumulation of protein complex that links the actin filaments from the last sarcomere to the ECM of the tendon cells.

The MTJ is a specific anatomical region that connects muscle and tendon [6] (Figure 1). This interface is generated through the integration of the extracellular matrix (ECM) of the tendon with the sarcolemma of skeletal muscle, forming a specialized and complex connection structure that provides huge mechanical strength to accommodate its function in transmitting mechanical forces [6]. Due to the critical role of the MTJ in force transmission, injury can lead to the loss of motor function and have significant economic and social impacts [10]. Therefore, gaining a comprehensive understanding of this intricate structure of the MTJ is of great significance.

Using optical microscopy, it can be seen that, at the MTJ in newborns, muscle fiber bundles insert into the dense connective tissue that forms the tendon [11]. The terminal ends of muscle fibers gradually thin out and split into narrow, brush-like protrusions, which interlace with the tendon collagen fibers [12]. Upon entering adolescence, the interface between muscle and tendon further intensifies, with the muscle cell membrane folding extensively to form finger-like protrusions and invaginations [11]. Mature muscle fiber terminals exhibit a serrated boundary, enabling tendon collagen fibers to penetrate deeply into the muscle fiber surface [12]. The formation of finger-like processes through the invagination and evagination of the membrane serves multiple physiological purposes. Firstly, it significantly enhances the contact surface between muscle and tendon, expanded by a factor of approximately 10–20 compared to a flat surface [10]. This increase in surface area effectively reduces stress, as stress is determined by the force divided by the area [10]. Secondly, it allows the membranes to be oriented at minimal angles as opposed to the vectors of applied force, resulting in the predominant exposure of the membranes to shear forces. Cell membranes commonly exhibit high resistance to shear stress, making this arrangement advantageous [10]. Some studies have indicated that, instead of the classic finger-like protrusions found in 2D TEM images, in 3D reconstructions of the MTJ, the tendon exhibits a prismatic projection intertwined with the recesses present in the muscle cell [13].

Under an electron microscope, the ultrastructure reveals that at the terminal muscle fiber of the MTJ, myofibrils branch widely at the last Z-disk and insert into the electron-dense layer known as the internal lamina, which corresponds to the position of the next Z-disk [10]. Subsequently, myofibers connect to the basal lamina through transmembrane proteins within the connecting domain and further associate with collagen fibers inside the tendon through extracellular crosslinking structures [6,14]. Trotter et al. proposed that the interface of the MTJ is composed of four distinguishable ultrastructural components that establish a connection between tendon collagen fibers and the terminal sarcomere [15].

In newborns, at the terminal end of muscle fibers in the MTJ, there are abundant clustered free ribosomes and polyribosomes, rich mitochondria, prominent Golgi complexes, and scattered endoplasmic reticulum in the cytoplasm, which are closely associated with the elongation of myofibrils and the development of the MTJ [16]. However, in the mature MTJ, particularly the Golgi complexes, these organelles are noticeably reduced in number. Dispersed mitochondria, smooth or rough endoplasmic reticulum, and localized accumulation of free ribosomes are commonly found at the subsarcolemmal regions of the MTJ. Leptomeric organelles are commonly observed beneath the muscle membrane in the MTJ of newborns and adolescents but are absent in the mature MTJ [16].

Previous research has yielded varied perspectives on the interfacial folding of the MTJ [6,17]. Some studies have proposed that the muscle infiltrates the tendon through finger-like protrusions, while others suggest that through MTJ finger-like or tubular invaginations, the tendon permeates the muscle cell surface. Electron micrographs provide evidence for both viewpoints, which may be influenced by the species or muscle type being examined [10]. Charvet proposes that the MTJ, originating from the primitive epithelial somite boundary, connects neighboring muscle cells, creating a mechanical module with the muscle [6]. However, Charvet’s definition does not encompass the sarcolemma and sarcoplasm of the MTJ [6]. According to Trotter, the MTJ represents a specialized area at the terminal skeletal muscle fibers with force transmission from intracellular myofibrils through the sarcolemma to the extracellular collagen fibers. The folding of the muscle–tendon interface is the most distinctive structural feature [15]. Ciena et al. define the MTJ as a highly specialized structural connection between muscle and tendon tissues, responsible for transmitting muscle-generated forces from myofibrils to the collagen fibers of the tendon [17].

In a clinical setting, a complete rupture at the MTJ can be easily diagnosed through physical examination and ultrasound. However, in cases of degenerative damage, partial tears, and other situations where the injury site is not clearly defined through ultrasound, magnetic resonance imaging (MRI) offers examiners a high-resolution field of view, providing a reliable method for diagnosing MTJ lesions [18]. Therefore, to accurately and comprehensively confirm the site of MTJ injury, clinical practice often involves a combination of methods such as physical examination, MRI, and specific muscle tests [19].

Therefore, we propose that a more accurate definition of MTJ could relate to its finger-like protrusions and their surrounding protruding areas. This is closely related to the function of the MTJ, which undertakes more mechanical stress by forming fascial folds through finger-like protrusions. In a broad sense, the region where the muscle and tendon intersect can also be referred to as the MTJ.

## 3. Subtle Structure and Characteristic Proteins of MTJ

**Figure 2:**
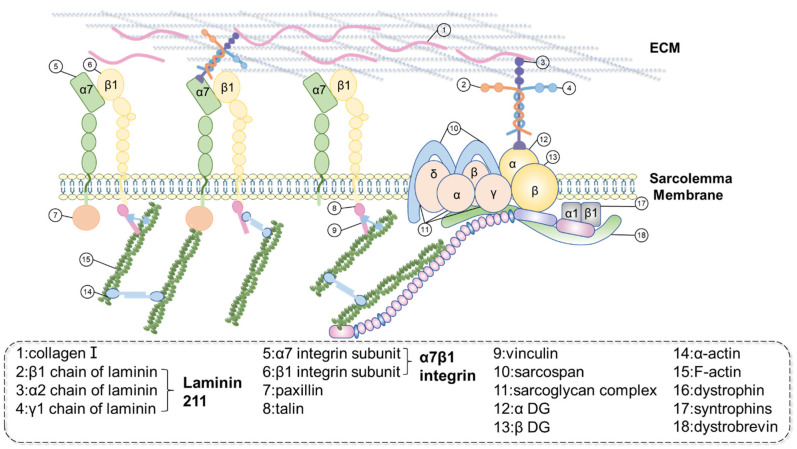
This figure shows a schematic diagram of the molecular level of the MTJ. Two types of protein complexes have been described so far: DGC (proteins 10–13 and 16–18) and a protein complex containing transmembrane α7β1 integrin (proteins 5–9). These complexes gather at the MTJ, corresponding to the sub-membrane density at the protrusions and link the sarcomeric actin to the ECM of the tendon cells through the basement membrane-associated protein α2, assembling into laminin 211 trimers. The two systems are linked through the intracellular protein α-actinin.

As described above, the subtle structure of the MTJ consists of the cytoskeleton system, connecting proteins, sarcolemma, and tendon components, including collagen I (Figure 2). The intensive connection of muscle and tendon is formed by several independent connecting systems with different components but serving similar functions, which link the filaments in muscle cells across the sarcolemma to the collagen in the tendon ECM. In this review, we highlight the pivotal roles of the dystrophin-associated protein complex (DAPC), also called the dystrophin–glycoprotein complex (DGC), and the α7β1 integrin-related system as the central connecting systems in the muscle–tendon interface. Additionally, we shed light on Col XXII, a key structural protein identified as a significant marker of the MTJ.

### 3.1. Dystrophin

In the MTJ, dystrophin plays a crucial role as a structural link between thin filaments and the sarcolemma [20]. Dystrophin, a monomeric 427-kDa cytoskeletal protein encoded by the X-chromosome located Duchenne muscular dystrophy (DMD) gene, is part of the β-spectrin/α-actinin protein family, which shares a common structural pattern, including an actin-binding domain at the NH2 terminus, followed by a variable number of spectrin-like repeats [21,22,23]. Dystrophin can be divided into four distinct regions: the central rod domain, the NH2-terminal actin-binding domain, the COOH-terminal domain, and the cysteine-rich domain [21]. The rod domain and the NH2-terminal actin-binding domain directly combine to actin without crosslinking [24]. The WW domain, dividing the rod domain from the cysteine-rich domain, mediates the coaction between dystrophin and β-dystroglycan by binding to the proline-rich domain of β-dystroglycan [24].

In terms of distribution, dystrophin is specifically situated near the surface of the sarcolemma in the MTJ [20]. Immunofluorescence indicates that the boundary of the distribution area of dystrophin is not clear, and the fluorescence intensity presents a gradual decrease in dystrophin intensity around the MTJ, extending towards the level of the sarcolemma outside of the junction [20].

Dystrophin is significantly associated with glycoprotein complexes that span the plasma membrane and have a binding affinity for laminin [22]. It has the function of supporting the mechanical integrity of the plasma membrane and/or participating in cytoskeleton–membrane interactions and is an important mechanical link connecting the cytoskeleton and membrane. Thus, dystrophin-deficient animals exhibit structural defects at the MTJ, with reduced lateral connections between thin filaments and the MTJ membrane and the inability of the muscle cytoskeleton to maintain connections with the membrane under physiological mechanical loading [20].

The DGC can be categorized into three categories: extracellular (α-dystroglycan), transmembrane (β-dystroglycan, sarcoglycans, and sarcospan), and cytoplasmic (dystrophin, dystrobrevin, syntrophins, and neuronal nitric oxide synthase) according to their cellular localization [25]. DGC plays a vital role in offering substantial protection to a portion of actin filaments, preventing their disassembly. Bassett et al. found that in DAPC-deficient mice, there was almost no folding at the MTJ, indicating that DAPC is required for normal MTJ formation. However, current research is still unclear about the specific effect of DAPC on MTJ [26].

### 3.2. α7β1 Integrin

Integrins, a diverse group of transmembrane heterodimeric receptors consisting of alpha (α) and beta (β) subunits, are widely distributed on the surface of vertebrate cells. Currently, at least twenty-four different α subunits and nine different β subunits have been recognized in humans. Integrins have significant functions in terms of cell adhesion, including interactions between cells and the ECM, as well as intercellular interactions [27]. Consequently, they participate in numerous biological processes, such as tissue repair and remodeling. When integrins cluster on the plasma membrane and respond to ECM ligands or anti-integrin antibodies, certain cytoskeletal proteins associate with integrins, resulting in the nucleation of actin filament bundles and assembling at specialized areas of cell–matrix adhesion [27].

The specificity of the location of integrin α7 at the MTJ may be attributed to its ECM ligand, which is considered laminin [28]. On the other hand, the interaction of α7 integrin with cytoskeletal components possibly guides its localization at the MTJ. Interactions between the cytoplasmic domain of the integrin β1 subunit and cytoskeletal-associated molecules have been reported [29].

The expression of α7 at the junction becomes apparent around embryonic day 14 and is predominantly expressed at the developing MTJ during this stage. The timing of the α7 subunit appearance in the MTJ is associated with the density of the myofiber inserted into the muscle layer and the folding of the connecting membrane, indicating the prominent role of the α7 integrin in MTJ development. The appearance of α7 occurs later than vinculin, the early marker of MTJ, indicating that α7 integrin can recognize the pre-existing MTJ cytoskeleton structure, and its localization is guided by the newly formed MTJ cytoskeleton. The selectivity of the presence of the α7 subunit in the MTJ suggests that α7 integrin is a determinant of connection specificity, aiding in further distinguishing the MTJ from other adhesive junctions and providing a molecular marker during MTJ development [30].

The lack of α7β1 in the MTJ exhibits significant ultrastructural defects, including muscle fiber end abrasion and compensatory changes in dystrophin localization, along with nearly absent MTJ folding. However, the overexpression of α7 integrin in the MTJ can restore the folding of the muscle layer, suggesting that α7 integrin functions significantly in maintaining the normal structure of the MTJ. As the MTJ is a vital region for force transfer, studies have found that α7 integrin-deficient mice show reduced grip strength in the forelimbs, indicating the importance of α7 integrin in daily force transmission from muscle to tendon. Therefore, α7β1 integrin is significant for the normal ultrastructure and force transmission of the MTJ [31].

### 3.3. Col XXII

Col XXII, expressed by muscle cells at tissue junctions, has been identified as a significant molecular marker for the MTJ. Immunofluorescence staining demonstrates the close proximity of Col XXII to the MTJ basal lamina, exhibiting a distinctive finger-like protrusion profile, and it has been shown that Col XXII interacts with α2β1 integrin expressing cells in vitro [32,33]. The partial co-localization of Col XXII with α11β1 integrin and α2β1 integrin at the MTJ has been revealed [34]. Through morpholino knockdown studies, it has been suggested that besides its interaction with laminin α2, Col XXII may bind to another laminin isoform and potentially interact with α7β1 integrin, while its primary involvement in the DGC appears limited [35].

Functional studies involving the knockdown of Col XXII expression in zebrafish have demonstrated that both larvae and adults exhibited deficiency in myoseptal structure, a significant reduction in MTJ folds, and various degrees of myofibers detachment [35,36]. These findings highlight Col XXII’s role in stabilizing MTJs and reinforcing muscle adhesion during contractive activity.

Recently developed single-nucleus RNA sequencing (scRNA-seq) has been used to gain some important insights into MTJ [37]. Dos Santos et al. performed scRNA-seq analysis of muscle fibers and found two distinct myonuclei types at the muscle ends and named them MTJ-A and MTJ-B [38]. The MTJ-A nuclei expressed genes known to be specifically expressed in the MTJ, such as Col22a1 and Itgb1, whereas the MTJ-B nuclei expressed a range of collagens known to be deposited in the MTJ, such as Col1a2, Col6a1, and Col6a3, and the major genes were expressed in connective tissue, such as Pdgfrb [38]. Unlike the MTJ-A type, MTJ-B type nuclei are not necessarily present in all myofibers and express myofiber and connective tissue markers [38]. Further analysis of this myonuclear subtype revealed that the MTJ-B subtype represents a population of myofiber nuclei that co-express myofiber and connective tissue-specific genes (Pdgfrb, Col6a3, and Ebf1) [38].

Anders Karlsen et al. conducted a proteomic analysis of human MTJs and identified 112 significantly enriched MTJ proteins, including 24 known MTJ-enriched proteins [39]. Immunofluorescence analysis confirmed the presence of tetraspanin-24 (CD151), kindlin-2 (FERMT2), cartilage intermediate layer protein 1 (CILP), and integrin-α10 (ITGA10) among 88 novel MTJ markers [39].

## 4. MTJ Injuries

In addition to traumatic injuries, injuries to the muscle–tendon interface often occur due to repeated overload, usually due to high-intensity training in young athletes or overuse in middle-aged and older adults [40]. While midsubstance tendon injuries are the most common injury, primarily affecting middle-aged patients, MTJ injuries are the second most common injury, affecting younger adults [41]. Patients with MTJ injuries rarely regain full pre-injury function. These diseases are difficult to control and often result in long-term pain and discomfort, placing a long-term burden on health care [9].

As the region between muscle and tendon, the structure of the MTJ determines its ability to transmit force longitudinally from muscle to tendon. In anatomical terms, the MTJ often includes the connective tissue surrounding the muscle bundle, which is continuous with the endomysium [10].

There are two distinct approaches for intramuscular force transmission, which can be characterized as follows: (1) a sequential transmission from sarcomere to sarcomere, ultimately connecting to the tendon (referred to as myo-tendinous transmission), and (2) a transmission from sarcomere to the myo-fascial complex, eventually leading to the tendon (known as myo-fascial to tendon transmission) [42].

The MTJ is prone to injury due to the mechanical disparities between tendon and muscle at the interface. When loads are applied to the muscle–tendon unit, homogeneous tension is generated across the region. However, each tissue (tendon and muscle) possesses different stiffness and cross-sectional area. As a result, the muscle, regarded as the thinnest and most compliant tissue at the tendon interface, bears the greatest strain and is more susceptible to failure, making the MTJ and nearby muscles vulnerable to damage [10,43].

Indirect trauma, which can be caused by exercise, stretching, and ischemia–reperfusion, is characterized by non-direct impacts on the muscle, while direct trauma, including muscle contusion and laceration, typically leads to tissue hemorrhage and edema [40].

**Figure 3:**
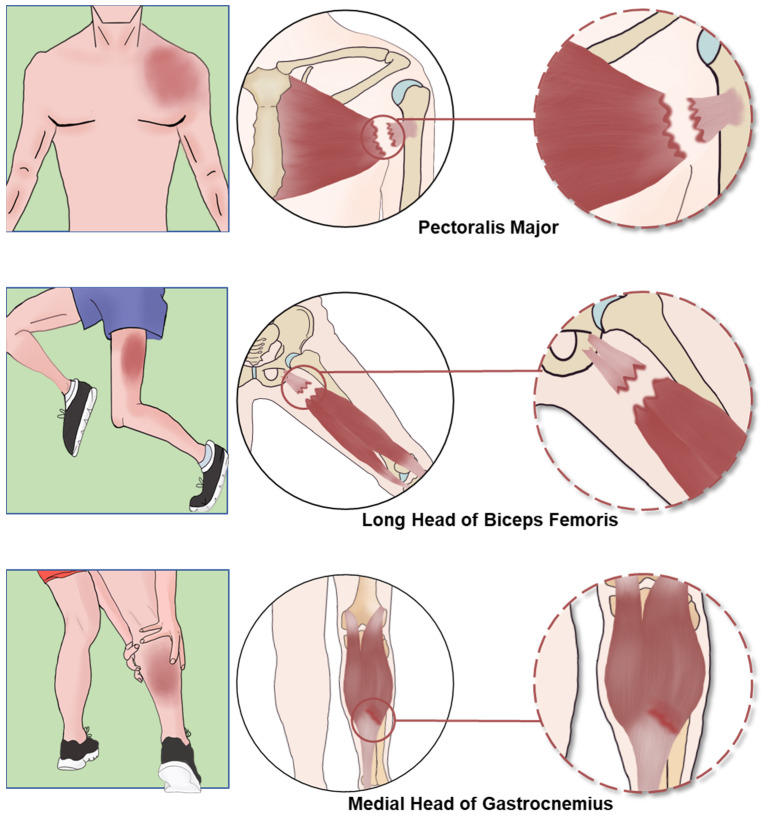
The picture illustrates the three common sites of MTJ injury: pectoralis major, gastrocnemius, and hamstring muscles. The PM muscle is prone to rupture during abduction and external rotation, and rupture often occurs near the distal MTJ. Distal gastrocnemius MTJ rupture, also known as “tennis leg”, is a relatively common MTJ injury caused by sudden overstretching of the muscle due to ankle dorsiflexing or knee extension. In addition, MTJ injuries often occur at the hamstring.

The MTJ is mainly affected by complete muscle tears [44]. MTJ injuries account for most bicep femoris injuries, about 14.4%. The supraspinatus has the highest incidence of tendon midsubstance injuries (11.4%). Complete tendon avulsions are more frequent in the triceps brachii and pectoralis major (1.1%) [41]. MTJ injuries often occur in the pectoralis major, gastrocnemius, and hamstring muscles (Figure 3) [45,46,47,48].

Clinical observations reveal that after a period of decreased muscle use, muscle tears, including those related to MTJs, usually appear during an acute episode of intense muscle activity, with MTJ tears frequently appearing under these circumstances during muscle eccentric loading [49].

In theory, failure could potentially originate at the MTJ but may propagate either through the MTJ itself or the adjacent muscle as the separation plane traverses the muscle. The exact location of failure during separation may depend on factors such as the activation state of the muscle cell, loading history, excessive MTJ breaking stress, strain rate, and the potential presence of prior injuries. Considering each of these variables is essential when discerning the conditions of strain injuries resulting in MTJ tears. The initiation of eccentric contraction-induced injury primarily originates from mechanical factors, and muscle tension assumes a vital role [50].

After MTJ injury, the most distant fibers near the MTJ exhibit ruptures and bleeding. Subsequently, histological analysis reveals pronounced inflammation, tissue edema, muscle fiber necrosis, leukocyte infiltration, and hemorrhage. Within 48 h, the damaged fibers experience complete rupture. Intense proliferation is exhibited in inflammatory cells, including macrophages, multinucleated cells, and fibroblasts. Macrophages invade muscle fibers, initially appearing undamaged, but they undergo significant injury during stretching. Subsequent phagocytosis and muscle regeneration follow a classic pattern described by many cell biologists in various models. Macrophages clear cellular debris, leaving behind the basal lamina, which functions as a scaffold for regenerating muscle fibers. After one week, inflammation significantly decreases, leukocytes become scarce, edema subsides, and bleeding ceases. Fibroblasts mature into elongated fibrocytes, with localized fibrosis visible at the injury site.

### Traditional Therapies for MTJ Injuries

Davide Curzi et al. studied the effects of exercise intensity on MTJ plasticity and on the expression of insulin-like growth factor 1 (IGF-1) and transforming growth factor beta (TGF-β) and their receptors in muscle and tendon [51]. MTJ complexity and interaction surfaces between tissues were increased in exercise-trained rats, and muscle strength was significantly enhanced in exercised rats [51]. In the muscle tissue of exercising rats, the mRNA of PGC-1α, vinculin, and TGF expression increased [11,51]. TGF-β mRNA expression was enhanced in the tendons of exercising rats, and Betaglycan tendon receptor mRNA levels were proportional to exercise intensity [11]. In conclusion, MTJ plasticity appears to be related to exercise intensity, and molecular analyses suggest that TGF-β plays a major role [11,51]. Therefore, a better understanding of the MTJ response to muscle exercise could improve injury prevention and allow for the planning of appropriate rehabilitation activities after MTJ trauma.

The most effective treatment options for acute MTJ injury include rest, icing, compression, and elevation, commonly known as the mnemonic “RICE” [52]. Resting in the short term helps reduce connective tissue proliferation at the region of MTJ injury, maximizing the healing response and restricting contraction [53]. In more severe cases of grade 2 and grade 3 MTJ injuries, crutches and bed rest are possibly necessary during the acute phase. Icing is widely accepted as the ultimate first-aid intervention for acute soft tissue injuries in the sports field. Ice has a physiological function on metabolism, inflammation, nerve conduction and circulation, most of which are beneficial for the healing process. Icing can delay inflammation and edema, thus limiting the spread of the MTJ injury area. Compression is believed to be effective in reducing bleeding, thereby limiting the inflammatory reaction and subsequent formation of scar tissue in the soft tissues. Another helpful effect may be the stimulation of proprioceptive feedback via a firmly applied compression bandage on the skin. The ultimate part of the treatment plan involves elevating the wounded body part above the level of the heart, which can effectively reduce swelling. Since inflammation is a pivotal feature of the body’s reaction to muscle injury caused by stretching, the most commonly accepted therapy is nonsteroidal anti-inflammatory drugs (NSAIDs). They work by inhibiting prostaglandin production. NSAIDs have little to no impact on other mediators involved in sustaining the ongoing inflammatory process. The anti-inflammatory effects of NSAIDs may be surpassed by their analgesic effects, enabling patients to recover activity faster and participate more efficiently in the recovery plan [52]. As pain and swelling subside, physical therapy should be started to restore flexibility and strength [54]. After a brief period of immobilization, mobilization is begun to properly align the regenerating muscle fibers and limit the extent of connective tissue fibrosis [52]. Concurrent pain-free stretching and strengthening exercises (beginning with isometrics and progressing to isotonics and isokinetics) are essential to regain flexibility and prevent further injury and inflammation [52].

Since pectoralis major, gastrocnemius, and hamstring muscles are usually involved in MTJ injuries [45,46,47,55]. The rupture of the medial head distal MTJ, commonly referred to as “tennis leg”, and the rupture of the pectoralis major (PMR) can be treated non-surgically. However, several studies have indicated that surgical treatment yields better effects in terms of function, strength, patient subjective ratings, and recovery to pre-injury performance [7,56,57,58]. Currently, the treatment for hamstring muscle injuries still remains controversial [59]. Increasing the strength of the hamstring muscles by lengthening their eccentric strength during loading and contraction is considered a method of preventing hamstring injuries [60,61]. Non-surgical treatment options commonly used for MTJ injuries in the pectoralis major and gastrocnemius muscles include rest, ice, elevation of the leg, and the use of pain medications and anti-inflammatories, followed by strengthening and stretching programs. However, during surgery, obvious anatomical damage is often found, and complete restoration can only be achieved through early surgical repair. Severe muscle tears treated conservatively can possibly heal with fibrotic scar tissue formation, which differs in its absorbency properties. This increases the risk of recurrent injuries [62]. Currently, indications for surgical intervention in gastrocnemius MTJ injuries include an inability to stand on tiptoes or bear weight solely on the affected leg’s metatarsal head, the inability to close through dorsiflexion and knee flexion, and severe (Grade III) tears or large intramuscular hematomas, especially when pain is accompanied by obvious defects and marked extension deficits. Early surgery allows for earlier functional recovery and leaves smaller defects [6,15]. For pectoralis major injuries, early surgical treatment yields better results [63]. Bak et al. confirmed the superior benefits of surgical treatment for PMR compared to non-surgical treatment [64]. Pochini et al. conducted a prospective randomized clinical trial and further proved the benefits of surgical repair for PMR [65]. Conservative treatment has been widely used in previous cases of hamstring muscle injuries and has shown good therapeutic effects [66]. However, several indications for surgical treatment are recommended, including (1) bone avulsion with a retraction of 2 cm, (2) complete tear of three tendons with or without retraction, and (3) symptomatic partial tears despite extensive conservative treatment [67,68,69].

In clinical practice, MTJ injuries are usually treated with traditional surgery. However, due to poor therapeutic effects and the complex interaction of the chemical, physical, and biological properties of the MTJ, increasing attention has been given to the use of tissue engineering in order to repair MTJ injury sites. With the continuous development of tissue engineering, new opportunities have been brought about in terms of MTJ regeneration.

## 5. Tissue Engineering Strategies for MTJ Injuries

Tissue engineering focuses on regenerating new tissue from cells with the support of biomaterials and growth factors [70]. Scott et al. used Hic1 to define quiescent mesenchymal progenitor cell subgroups with distinct functions and fates during skeletal muscle regeneration [71]. They believed that Hic1 regulates the quiescence of a subset of mesenchymal progenitor cells and that Hic1+ progenitor cells can promote MTJ regeneration after trauma [71]. Yassin et al. discovered connecting cells with dual characteristics from MTJ and explored the potential mechanism of MTJ-centered musculoskeletal system construction [72]. Ruojin Yan et al. analyzed the cell subtype distribution and function of human MTJ at the single-cell level and discovered muscle–tendon progenitor cells (MTPs) [5]. The MTP subpopulation retains the characteristics of stem cells and expresses muscle and tendon marker genes at the same time, which may have the potential for bidirectional differentiation. Moreover, the MTP showed strong regeneration ability after transplantation into the MTJ injury model, laying the foundation for the research on regeneration and repair of myotendinous junctions [5].

Utilizing scaffold-based tissue engineering strategies provides an innovative approach to addressing MTJ damage [73]. However, fully mimicking the unique characteristics of muscles, tendons, and the MTJ itself to custom scaffolds for the MTJ is a challenging task due to the complex interplay of biological, chemical, and physical properties at the MTJ [74]. Nonetheless, some studies have shown the potential of various scaffolds in promoting the effective repair of MTJ injuries (Table 1).

### 5.1. Biological Scaffold

Biological scaffolds that have been applied in MTJ tissue engineering include decellularized ECM scaffolds and hydrogel scaffolds [75,82,83]. Decellularized ECM scaffolds are processed through a series of steps, including lipid removal, cell destruction and sterilization, to remove any non-collagen components in mammalian tissues that may cause host rejection, with the preservation of mechanical properties and natural collagen structures [83]. Turner et al. investigated the capability of ECM-based scaffolds to promote the recovery of distal gastrocnemius muscle MTJ function in a canine model after complete tissue excision [75]. The results of the study indicated the formation of vascularization and neural innervation in the skeletal muscle at the region of ECM scaffold implantation that resembled normal muscle tissue [75]. ECM hydrogels allow for cell suspension within the matrix, creating a more suitable microenvironment for cell attachment and spreading compared to monolayer culture. Gaffney et al. prepared tissue-specific hydrogels from decellularized muscle and tendon tissues to mimic the composition of the tissue microenvironment [82]. Cells cultured in ECM hydrogels exhibited increased expression of MTJ-specific paxillin protein and Col XXII [82]. However, considering the xenogeneic nature of biological scaffolds, they may induce pro-inflammatory reactions upon implantation [84]. Fibrosis or scar tissue formation may occur when resident cells in the source tissues have not been completely eliminated [84,85,86]. Therefore, it is necessary to thoroughly remove resident cells through methods such as lipid or fat deposition removal, cell destruction and sterilization [74]. Once resident cells have been effectively removed through proper processing, the biological scaffolds can induce a macrophage response, thereby correctly guiding the pro-inflammatory reaction [74,84]. Type I collagen within the scaffold possesses bioactivity and can promote cell proliferation and tissue ingrowth [73,74]. Thus, the biological scaffold is relatively straightforward in principle. However, the use of allogeneic or xenogeneic scaffolds may carry risks of disease transmission and immune rejection without the complete elimination of cells and non-ECM materials [87].

### 5.2. Synthetic Scaffold

In tissue engineering, scaffolds derived from synthetic polymers are called synthetic scaffolds. Due to their flexible material properties, synthetic polymers are attracting attention [88,89]. Synthetic scaffolds are characterized by nontoxicity, low production costs, tunable and replicable mechanical and chemical properties, and are easily molded into various forms [77,78]. Generally, synthetic scaffolds do not pose a risk of disease transmission [74]. The ideal synthetic polymer scaffold must guide the interaction between heterogeneous and homogeneous cells, with the function of promoting the maintenance and the formation of controlled matrix heterogeneity [77]. Therefore, the scaffold is supposed to exhibit morphology and specific morphological gradients similar to the insertion sites of the native tissue [90].

Electrospinning is a commonly used technique for designing MTJ structures [9]. Nanofiber-based nonwoven mats made via electrospinning are small in size and typically exhibit high porosity and a larger surface area [9]. Therefore, they can mimic the hierarchical structure of the ECM, which is highly relevant to cell adhesion and nutrient transport [79]. Fibers can also be encapsulated or attached to bioactive substances [80]. Ladd et al. produced an electrospun scaffold featuring a flexible/high-strain region, a rigid/low-strain region, and a middle region, which overcame the challenge of matching different mechanical properties between muscle and tendon [91]. However, the scaffold proposed in this study is not without its limitations: its mechanical performance does not match that of the native MTJ despite the fact that the performance trend is similar between the scaffold and the native MTJ [91]. The entire scaffold has higher hardness and ultimate tensile strength (UTS) than the native MTJ but lower strain at failure. Additionally, cyclic testing indicates that the current form of the scaffold may not be perfect as it undergoes creep after a relatively small number of cycles compared to the MTJ structure [91].

Although electrospinning is applied in the regeneration of the MTJ [9], the electrospinning structures are discontinuous, resulting in an array of separated fibers, and the subsequent issue is the mechanical integrity of the generated scaffold. Freeze-drying and gel-drying hydrogels have been posed for tissue engineering relative to the MTJ in some limited cases, utilizing collagen and other polymeric materials to generate the concerned portions of the natural ECM [92]. Gel drying possibly functions well in MTJ regeneration when mineralization gradients are applied along the structure. Unlike electrospinning, it starts from a homogeneous (single block) structure incorporating typical gel and hydrogel nanostructured fibers [93]. From a macroscopic perspective, gels can be organized into any shape. Additionally, hydrogels require crosslinking to produce stable (non-water-soluble) structures, and their partial performance may be compromised. Crosslinking agents, such as glutaraldehyde, have cytotoxicity; thus, their accurate elimination is also necessary [94,95]. Three-dimensional printing provides another way of thinking about the basic organizational structure of the MTJ [8,96]. Merceron et al. employed bioprinting to fabricate thermoplastic polyurethane (PU) and C2C12 myoblast cells for the muscle side, as well as polycaprolactone (PCL) and NIH/3T3 fibroblast cells for the tendon side. A fibrous protein-based hydrogel ink was crosslinked to create interfaces between PU and PCL [81]. The resulting structure demonstrated elasticity on the PU-C2C12 muscle side, rigidity on the PCL-NIH/3T3 tendon side, and intermediate hardness in the interface site [81]. Furthermore, both cell lines exhibited proper growth on their respective surfaces, and some interface features were found. With the relatively easy establishment, this type of approach appears promising [81]. The next procedure would involve the use of more relevant cell types and the application of induced mechanical stretching stimuli to exert pressure on three regions exhibiting distinct mechanical properties in order to generate different mechanotransduction signals.

## 6. Conclusions

This review provides a detailed introduction to the MTJ and representative therapeutic methods in MTJ tissue engineering, aiming to contribute more ideas to the development of MTJ tissue engineering. The MTJ is a highly specialized tissue interface at the junction between muscle and tendon, which is significant for force transmission. This review discusses the definition, anatomical structure, unique structural proteins at the MTJ, its functions, injuries, and repair. It summarizes the traditional definition of the MTJ from clinical and anatomical perspectives and proposes a precise definition of the MTJ. Integrin α7β1, DGC and Col XXII are among the specific components found at the MTJ. Due to the continuous mechanical stress on the MTJ, it is prone to injury. Current treatment options include compression ice therapy, medication, and surgical intervention. The advantage of non-surgical treatment is safety and simplicity, but it is inferior to surgical treatment in terms of returning patients to preoperative results. However, surgical treatment has higher risks and postoperative recurrence rates. This review summarizes representative advancements in MTJ tissue engineering, focusing on scaffolds. It introduces therapeutic approaches for the MTJ using biological and synthetic scaffolds and highlights the commonly used technique of electrospinning for constructing MTJ scaffolds. The use of scaffold-based tissue engineering strategies has the potential to promote the effective repair of MTJ injuries, but creating a scaffold that fully simulates the MTJ is challenging. Based on the delicacy and complexity of the MTJ structure, MTJ injury can lead to muscle and tendon damage, resulting in dysfunction of the motor system. The current challenge is to explore more submicroscopic structures of the MTJ and the factors that affect MTJ injury and regeneration. In addition, current research on MTJ is mainly based on animals. Future MTJ research can focus more on humans and explore the function and repair of human MTJ. We hope that in the future, we can thoroughly understand the molecular signals of MTJ in force transmission, optimize the traditional surgical treatment of MTJ, and innovate MTJ tissue engineering technology to promote the repair and regeneration of MTJ.

## Figures and Tables

**Table 1:** The main tissue engineering techniques associated with MTJ.

Techniques	Advantages	References
Decellularized ECM scaffolds	Mimicking a non-immune environment with native three-dimensional structures and various bioactive components	[74,75,76]
Hydrogel scaffolds	Nontoxicity, low production costs, tunable and replicable mechanical and chemical properties	[77,78]
Electrospinning	High porosity and a larger surface area, promoting cell adhesion and nutrient transport	[79,80]
3D printing	Easy to create, simulating muscle and tendon interfaces	[81]
